# Differences in acute physiological response to a Qigong exercise among middle-aged adults with varying durations of Qigong practice

**DOI:** 10.3389/fphys.2025.1699846

**Published:** 2026-01-08

**Authors:** Jingyu Sun, Zhangxiaohe Zhang, Rongji Zhao, Nannan Jia, Jiajia Chen, Duran Qin, Jing Wang, Zhengyi Zhang, Hao Yang, Tianfeng Lu, Antonio Cicchella, Tao Chen

**Affiliations:** 1 Sports and Health Research Center, Tongji University, Shanghai, China; 2 Department of Physical Education, Wuhan Business University, Wuhan, China; 3 Department for Quality-of-Life Studies, Bologna University, Bologna, Italy

**Keywords:** heart rate variability, middle-aged adults, physical fitness, Qigong, respiration

## Abstract

**Background:**

Qigong combines physical movement, breath control, and mental focus, offering potential benefits for healthy aging. Since physiological decline begins in midlife, this stage is critical for preventive intervention. The purpose of this study was to investigate the differences in acute physiological responses, including autonomic (heart rate variability) and respiratory indicators, during a single session of combined Yijinjing and Liuzijue Qigong in middle-aged adults with varying durations of practice experience (≥4 years vs. ≤6 months).

**Methods:**

Forty adults aged 40 to 59 were included and divided into two groups based on Qigong exercise experience: an experienced group (*n* = 20) with ≥4 years of practice, and an inexperienced group (*n* = 20) with <6 months. All participants performed 20 min of fitness Qigong, following prerecorded tapes to standardize pace and posture sequence. Heart rate variability (HRV) and respiration were measured using the Biofeedback 2000x-pert system before, during, and after the session.

**Results:**

Significant Group × Time interactions were found for SDNN (F = 5.58, *p* = 0.012), RMSSD (F = 20.52, *p* < 0.001) and LF (F = 5.47, *p* = 0.025). Between-group comparisons indicated that experienced group had slightly higher SDNN at rest (*p* = 0.039) and significantly higher RMSSD during the recovery phase (*p* < 0.001); no other between-group differences emerged at other phases. There was a significant Group × Time interaction for abdominal breathing depth (F = 3.911, p = 0.024) and thoracic breathing frequency (F = 4.956, p = 0.016). Between-group comparisons revealed deeper abdominal breathing during exercise and slower thoracic breathing during recovery in the experienced group.

**Conclusion:**

Middle-aged adults with prolonged practice of Qigong exercise have improved HRV compared to those in the inexperienced group, and these improvements may be achieved through a combination of breathing adjustments with mental focus and relaxation.

## 1 Introduction

With the rapid growth of the aging population and the escalating costs of healthcare, promoting health and preventing disease during adulthood, particularly from middle age onward, has become a critical focus for public health initiatives (Gianfredi et al., 2025). Middle age represents a pivotal stage in the aging trajectory, as functional decline often begins during this period, including gradual deterioration in the cardiovascular, respiratory, and neuromuscular systems (Motanova et al., 2024). Therefore, initiating preventive interventions in midlife may offer an effective strategy for promoting healthy aging and delaying age-related decline.

Regular exercise is essential for healthy aging and offers many health benefits, including reduced risks of all-cause mortality, chronic disease, and premature death (Jones et al., 2024). For older adults, exercise recommendations emphasize aerobic activity, muscular strength, and flexibility (Jones et al., 2024). Among the various forms of exercise, Qigong, a traditional Chinese mind-body practice, has gained increasing recognition as a low-impact, accessible form of activity suitable for aging populations (Zeng et al., 2024).

Qigong integrates gentle physical movements with controlled breathing, meditative focus, and heightened body awareness, offering potential benefits across multiple physiological domains (Antonelli and Donelli, 2024). Studies have reported improvements in balance, flexibility, respiratory function, and autonomic regulation through Qigong practice (Wang Z. et al., 2024). Specific forms such as Yijinjing focus on musculoskeletal health through dynamic postural changes, while Liuzijue emphasizes breath control and vocalization to enhance respiratory function (Xu et al., 2022). Combining these two forms may provide a comprehensive approach to improving physical function in middle-aged adults.

Despite the growing popularity of Qigong as a meditative exercise, research has primarily focused on its short-term effects or subjective outcomes (Mazzocco et al., 2023; Zhang Y. P. et al., 2020), and the long-term physiological adaptations associated with continued practice remain underexplored. In particular, it is unclear whether individuals with extended experience in Qigong training demonstrate more favorable physiological responses, such as improved autonomic regulation and respiratory performance, compared to beginners.

Heart rate variability (HRV) and respiratory function are physiological indicators because they are critical to healthy aging (Yelisyeyeva et al., 2025). HRV reflects the adaptability and resilience of the autonomic nervous system, while efficient respiratory function supports cardiovascular health and metabolic balance (Olivieri et al., 2024; Zhang W. et al., 2025). Both are closely linked to age-related decline (Agorastos et al., 2023; Zhang W. et al., 2025) and are potentially modifiable through regular mind-body exercise such as Qigong. Therefore, the purpose of this study was to investigate the differences in acute physiological responses, including HRV and respiratory indicators, during a single session of combined Yijinjing and Liuzijue Qigong in middle-aged adults with varying durations of practice experience (≥4 years vs. ≤6 months). Understanding these differences may offer insights into the physiological mechanisms underlying Qigong’s health benefits and inform future strategies for exercise-based health promotion in aging populations.

## 2 Methods

### 2.1 Participants

Practitioners aged 40–59 years were recruited for this study. The inclusion criteria were as follows: (1) age between 40 and 59 years; (2) no apparent limitations to daily activities; (3) either over 4 years of experience in fitness Qigong or no more than 6 months of experience if engaged in other regular aerobic exercise; (4) unrestricted exercise capacity, a body mass index (BMI) between 18.5 and 24.9, and the ability to follow instructions; and (5) provision of informed consent. Based on their training history and competitive achievements (as assessed by experts), the participants were categorized into two groups: experienced practitioners (≥4 years of fitness Qigong experience) and inexperienced practitioners (≤6 months of fitness Qigong experience).

The exclusion criteria were: (1) a medical condition (e.g., neurological, orthopedic, or rheumatic disorders): that could impair the performance of mobility tasks; (2) a history of heart disease, severe arrhythmia, or use of a pacemaker; (3) smoke addiction or frequent heavy consumption of alcohol and other stimulating beverages; and (4) use of medications known to influence autonomic functions (e.g., antidepressants, antihypertensives, or anticholinergics) within 1 month prior to the study.

**FIGURE 1:**
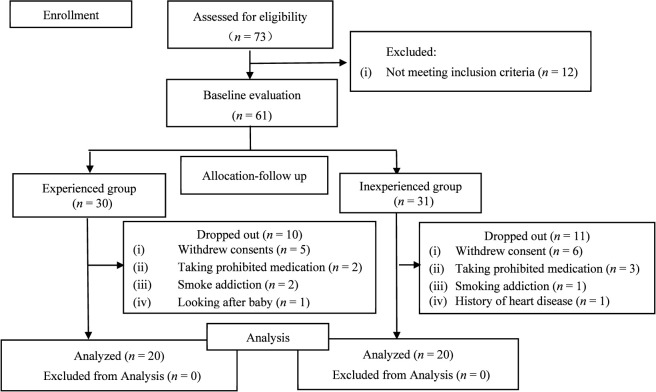
Participants flow diagram.

This study initially enrolled 61 practitioners (30 experienced and 31 inexperienced). In the experienced group, 10 participants dropped out for the following reasons: withdrawal of consent (*n* = 5), use of prohibited medication (*n* = 2), smoking addiction (*n* = 2), and personal reasons (*n* = 1). In the inexperienced group, 11 participants dropped out for the following reasons: withdrawal of consent (*n* = 6), use of prohibited medication (*n* = 3), smoke addiction (*n* = 1), and a pre-existing health condition (*n* = 1). Consequently, the final analysis included 40 participants, with 20 in each group (Figure 1).

### 2.2 Experimental procedures

The participants were requested to refrain from exercising or drinking alcoholic or caffeinated beverages for at least 24 h before the test. All participants were tested individually. The participants were required to read and sign informed consent forms when they arrived at the test site. All participants were required to complete basic screening forms and questionnaires to assess their health status, life events, and cognitive function before the conduct of the experiment. Subjects performed fitness Qigong exercises for 20 min and were asked to follow prerecorded tapes to ensure that they had the same pace and sequence of postures. HRV (standard deviation of NN intervals [SDNN], root mean square of the successive differences [RMSSD], low frequency [LF], high frequency [HF], low frequency/high frequency ratio [LF/HF]), respiration depth and frequency data (the maximum and minimum of abdominal respiration curve, the maximum and minimum of thoracic respiration curve, the number of abdominal breaths per minute (which is calculated from the abdominal respiration curve, and the number of thoracic breaths per minute, (which is calculated from the thoracic respiration curve)were collected in three periods: (1) 10 min of rest in a chair before the start of exercise, (2) 20 min of exercise with instrument, and (3) 10 min of recovery period (sitting position) after the exercise. Trends in HRV and respiratory index were recorded with the instrument and transferred to a computer for data analysis. The entire process was conducted between 14:00 and 17:00 at 24 °C–26 °C in a constant-temperature and bright lounge. All instruments used in the experiments were operated by experienced technical personnel. In all cases, the anonymity and confidentiality of their data were guaranteed. Subjects gave their informed consent for inclusion before they participated in the study. The study was approved by the Ethics Committee of Tongji University (2021tjdx024).

### 2.3 Fitness Qigong movement

The fitness Qigong movements of Yijinjing and Liuzijue that are targeted for middle-aged people were implemented in this study. The movements combine meditation, relaxation, and gentle movements with breathing. The movement patterns of the two groups were identical. The 12 movements of Yijinjing are aimed at strengthening musculoskeletal fitness and circulation and are performed along with breathing training. Liuzijue is an art of expiration that can produce six different sounds (Xu, He, Hu, Si, Chui, and Xi). The participants were required to coordinate their breathing with the prescribed movements. The six types of Liuzijue pronunciation correspond to different internal organs, including outgoing “Xu” to protect the liver and waist, “He” to keep the heart vigorous, “Hu” to invigorate the spleen and nourish the stomach, “Si” to moisten the lungs, “Chui” to guard the kidneys, and “Xi” to tonify qi pulse. These sound exercises may result in increased asynchronous and paradoxical breathing movements.

### 2.4 Measurements

#### 2.4.1 Body mass index, basal metabolic rate

The body mass index (BMI) and basal metabolic rate (BMR) of the participants were measured using MC-980MA (Tanita, Japan).

#### 2.4.2 36-Item Short Form Survey instrument

The SF-36 questionnaire was used to assess the participants’ quality of life. This multidimensional questionnaire consists of 36 items subdivided into eight domains to measure functional health, sociality, and emotion from the participants’ perspectives (Brazier et al., 1992). Item 1 is not calculated for any domain but is only used to assess the general health status of participants. The final score ranges from 0 to 100, and higher scores indicate better physical and mental health.

#### 2.4.3 HRV measurement

HRV was assessed using a 2000x-pert biofeedback instrument (Biopac Systems Inc.), a modular and portable device for monitoring physiological parameters. Sensor signals within its radio modules were filtered, amplified, digitized, and transmitted via Bluetooth to a computer. The electrocardiogram (ECG) data were processed using peak-detection algorithms to locate R-waves, in accordance with the manufacturer’s instructions (Fiľo and Janoušek, 2022; Lee M. S. et al., 2005). Care was taken to minimize motion artifacts and DC interference to ensure signal quality sufficient for data processing.

Data were collected during three distinct phases: before (rest), during (exercise), and after (recovery) the fitness Qigong movements. For a consistent longitudinal comparison, stable 5-min segments were extracted from each of these three periods and processed anew using Biofeedback Xpert software for HRV analysis (Duan et al., 2025). HRV was evaluated in the time and frequency domains. The time-domain indicators included SDNN and RMSSD. The frequency domain included raw LF (0.04–0.15 Hz), HF (0.15–0.4 Hz), and the LF/HF ratio.

#### 2.4.4 Respiration measurement

Respiratory indices, including the amplitude and frequency of thoracic and abdominal breathing, were assessed using the Biofeedback 2000x-pert system (Biopac Systems Inc.). Respiratory measurements included four indicators: (1) Depth of Abdominal Respiration; (2) Frequency of Abdominal Respiration; (3) Depth of Thoracic Respiration; and (4) Frequency of Thoracic Respiration. Respiration straps were placed at the thoracic and abdominal areas and pressed into the groove on the module. The RESP module was calibrated in the middle range at the start of every session to improve the accuracy of the experiments. The participants were required to breathe naturally during the period of pre- and post-movement and coordinate their breathing with their body movements during the fitness Qigong exercise.

### 2.5 Sample size

The sample size was calculated based on the primary outcome of HRV, specifically SDNN. A recent meta-analysis examining the effects of Tai Chi and Qigong on HRV parameters in adult populations reported a standardized mean difference (SMD) of 0.83 for SDNN, indicating a large effect size compared with control conditions (P = 0.02; 95% CI [0.16, 1.51]) (Larkey et al., 2024). Using this effect size (Cohen’s f ≈ 0.415) in G*Power for a repeated measures ANOVA with a group × time (2 × 3) design, an alpha level of 0.05, and power of 0.80, the required sample size was estimated to be 24 participants total (12 per group). To account for potential attrition (∼15%), we aimed to recruit 40 participants (20 per group).

### 2.6 Statistical analysis

SF-36 scores between the two groups were compared via an independent sample t-test. A repeated-measures ANOVA was used to examine the main effects (group and time) and the interaction effects (group*time) on the independent variables. Bonferroni’s test was used for *post hoc* analysis to compare the differences between groups and times. Data are expressed as mean ± SD. Statistical significance was accepted if the P-value was <0.05. The partial eta square is used to represent the effect size (ES). The statistical package IBM SPSS Statistics software (SPSS) was used for statistical analysis.

## 3 Results

### 3.1 Demographic characteristics and SF-36 score of the two groups of participants

The first part of Table 1 shows the basic characteristics of the participants across the two groups. The age of the participants was between 40 and 59 years. The average height, weight, BMI, and BMR of the two groups were similar. The experienced group scored higher than the inexperienced group in role physical, general health, and emotional role functioning domains (*p* < 0.05). Although no significant differences were observed in the other five domains, the scores of the experienced group were slightly higher than those of the inexperienced group.

**TABLE 1:** Characteristics and SF-36 scores (*n* = 40).

Variable	Characteristics/Domain	Inexperienced (*n* = 20)	Experienced (*n* = 20)
Demographic characteristics	Gender (M&F)	75% & 25%	65% & 35%
	Age (year)	50.49 ± 9.68	52.6 ± 7.2
	Height (cm)	162.03 ± 7.02	161.4 ± 5.3
	Weight (kg)	58.35 ± 4.85	60.63 ± 2.02
	BMI (kg/m2)	25.49 ± 2.14	23.2 ± 0.70
	BMR (KJ)	1297.90 ± 68.34	1287.67 ± 28.60
	HR (times/min)	73.69 ± 8.87	75.40 ± 7.53
	Education level (bachelor degree and postgraduate)	55%	80%
SF-36 domain	Physical functioning	84.65 ± 5.73	89.07 ± 9.72
	Role physical	67.07 ± 29.15	81.57 ± 15.13*
	Bodily pain	72.42 ± 17.06	78.07 ± 8.06
	General health	50.67 ± 6.68	64.75 ± 7.44*
	Vitality	71.27 ± 16.73	77.03 ± 9.71
	Social role functioning	83.75 ± 17.51	92.02 ± 6.27
	Emotional role functioning	53.08 ± 34.47	66.36 ± 36.02*
	Mental health	71.89 ± 11.57	72.82 ± 7.72

Values represent mean ± SD.

* represent a statistically significant difference between two groups (p < 0.05).

### 3.2 Effects of fitness Qigong exercise on HRV indexes in middle-aged adults

Table 2 summarizes group differences in HRV indices across the three experimental phases. A significant Group × Time interaction was found for SDNN (F = 5.58, *p* = 0.012), RMSSD (F = 20.52, *p* < 0.001), and LF (F = 5.47, *p* = 0.025). Post-hoc between-group analyses showed that the experienced group had slightly higher SDNN at rest (*p* = 0.039) and significantly higher RMSSD during recovery (*p* < 0.001), while no group differences were observed at other phases. Within both groups, SDNN, RMSSD, and LF decreased markedly from rest to exercise and rebounded during recovery (all *p* < 0.001). The experienced group consistently exhibited greater decreases during exercise and larger rebounds during recovery compared to the inexperienced group. For example, within the experienced group, SDNN significantly decreased from rest to exercise (−87.3 ± 4.15 m, *p* < 0.0001), and then increased substantially from exercise to recovery (+80.5 ± 3.58 m, *p* < 0.0001). The SDNN in the inexperienced group displayed a similar pattern, but with smaller changes (−71.3 ± 4.15 m, +70.2 ± 3.58 m; both *p* < 0.0001). No significant Group × Time interactions, but main time effects were detected for HF and the LF/HF ratio. Across all participants, HF declined from rest to exercise and partially recovered post-exercise, whereas LF/HF increased during exercise and decreased during recovery (all *p* < 0.001). Moreover, the main group effect was also observed for the LF/HF ratio.

**TABLE 2:** Differences in HRV measurements between the experienced and inexperienced groups.

Variable	Period	Experienced (n = 20)	Inexperienced (n = 20)	Source	F	p
SDNN (ms)	Resting	146.01 ± 15.22*	136.22 ± 13.72	Group	0.66	0.423
	Exercise	58.75 ± 13.37^&&&^	64.90 ± 14.33^&&&^	Time	683.55	<0.001
	Recovery	139.25 ± 12.14^##,^ ^^^^^	135.10 ± 11.51^^^^^	G*T	5.58	0.012
RMSSD (ms)	Resting	55.12 ± 3.08	53.18 ± 3.20	Group	13.11	<0.001
	Exercise	25.19 ± 2.86^&&&^	24.63 ± 1.61^&&&^	Time	2174.6	<0.001
	Recovery	46.10 ± 3.49*** ^,###,^ ^^^^^	40.03 ± 3.26*** ^###,^ ^^^^^	G*T	20.52	<0.001
LF (ms^2^)	Resting	313.39 ± 32.59	314.55 ± 31.15	Group	0.04	0.853
	Exercise	299.55 ± 33.46^&&&^	302.54 ± 31.52^&&&^	Time	984.97	<0.001
	Recovery	306.47 ± 33.00^###,^ ^^^^^	308.04 ± 31.30^###,^ ^^^^^	G*T	5.47	<0.025
HF (ms^2^)	Resting	276.47 ± 37.25	259.57 ± 36.39	Group	1.02	0.32
	Exercise	196.32 ± 24.47^&&&^	191.04 ± 17.34^&&&^	Time	244.37	<0.001
	Recovery	220.91 ± 24.46^###,^ ^^^^^	219.50 ± 19.13^###,^ ^^^^^	G*T	2.8	0.09
LF/HF	Resting	1.14 ± 0.08**	1.22 ± 0.09	Group	20.5	<0.001
	Exercise	1.53 ± 0.06* ^,^ ^&&&^	1.58 ± 0.05^&&&^	Time	276.08	<0.001
	Recovery	1.39 ± 0.06^###,^ ^^^^^	1.40 ± 0.05^###,^ ^^^^^	G*T	2.19	0.129

*p < 0.05, **p < 0.01, ***p < 0.001 comparison of the same index between two groups in the same moment.

&&& *p <* 0.001 comparison between resting and exercise states.

^^^ p < 0.001 comparison between recovery and exercise states within each group. SDNN, the standard deviation of R-R intervals; RMSSD, root mean square of the successive differences; LF, low-frequency power; HF, high-frequency power; LF/HF, low-frequency/high-frequency ratio.

### 3.3 Effects of fitness Qigong exercise on breath index in middle-aged adults

Table 3 presents group differences in respiration patterns across the three phases. For abdominal respiration depth, a significant group-by-time interaction was found (F = 3.911, *p* = 0.024). Post-hoc analysis revealed that the experienced group exhibited deeper breathing during exercise (*p* < 0.001), compared to the inexperienced group. Thoracic respiration depth showed no significant interaction (F = 0.992, *p* = 0.349). However, the experienced group had a consistently greater overall amplitude than the inexperienced group (main effect of group: F = 5.838, *p* = 0.021). Both groups demonstrated increased thoracic amplitude during exercise and partial normalization in recovery (main effect of time: F = 131.546, *p* < 0.001). For abdominal respiration frequency, no significant interaction was observed (F = 1.443, *p* = 0.244). Nonetheless, the experienced group consistently breathed more slowly across all phases (main effect of group: F = 14.267, *p* < 0.001), and both groups showed an increase in frequency during exercise followed by a return toward baseline in recovery (main effect of time: F = 95.292, *p* < 0.001). Finally, thoracic respiration frequency exhibited a significant group-by-time interaction (F = 4.956, *p* = 0.016). The experienced group had a slower breathing rate at rest, during exercise, and particularly in recovery (*p* = 0.016).

**TABLE 3:** Differences in respiration measurements between the experienced and inexperienced groups.

Variable	Period	Experienced. (n = 20)	Inexperienced (n = 20)	Source	F	p
Depth of abdominal respiration (cm)	Resting	0.66 ± 0.20	0.61 ± 0.24	Group	8.072	0.007
	Exercise	1.30 ± 0.36* ^,^ ^&&&^	0.97 ± 0.21^&&&^	Time	53.817	<0.001
	Recovery	0.84 ± 0.34^#^ ^,^ ^^^^^	0.67 ± 0.24^^^^^	G*T	3.911	0.024
Depth of thoracic respiration (cm)	Resting	0.63 ± 0.19	0.52 ± 0.10	Group	5.838	0.021
	Exercise	1.20 ± 0.41^&&^ ^,^ ^^^^^	1.12 ± 0.18^&&^	Time	131.546	<0.001
	Recovery	0.71 ± 0.21* ^,^ ^#^	0.52 ± 0.14^^	G*T	0.992	0.349
Frequency of abdominal respiration (ventilations/min)	Resting	14.16 ± 2.55	16.33 ± 2.39	Group	14.267	<0.001
	Exercise	21.65 ± 3.27^&&&^	24.18 ± 4.69^&&&^	Time	95.292	<0.001
	Recovery	13.77 ± 3.91* ^,^ ^^^^^	17.92 ± 2.62^^^	G*T	1.443	0.244
Frequency of thoracic respiration (ventilations/min)	Resting	14.84 ± 1.77	16.48 ± 2.06	Group	8.157	0.007
	Exercise	22.45 ± 3.06^&&&^	23.16 ± 4.19^&&&^	Time	129.095	<0.001
	Recovery	14.77 ± 2.61* ^,^ ^^^^^	18.45 ± 2.68^##^ ^,^ ^^^^^	G*T	4.956	0.016

* p < 0.05, comparison of the same index between two groups in the same moment.

^&&^p < 0.01, ^&&&^p < 0.001 comparison between resting and exercise moments.

^#^p < 0.05, ^##^p < 0.01, comparison between resting and recovery moments.

^^^ *p <* 0.001 comparison between recovery and exercise moments within each group.

## 4 Discussion

This study was designed to examine the effects of different years of fitness Qigong training on the HRV and respiration function in middle-aged adults. Results demonstrated that prolonged duration of Qigong exercise causes a significant improvement in HRV. These benefits can potentially be achieved in part through a combination of breathing regulation with mental focus and relaxation.

To date, few trials have investigated the effects of fitness Qigong on HRV and breath regulation to support age-related physical fitness. However, some studies showed that regular Qigong Baduanjin style exercise not only delays normal age-related decline in the memory domain but also lowers blood pressure and blood lipids and improves quality of life, fatigue, and emotional state, and its effect is better than that of jogging (An-Li, 2008). Physical fitness is reportedly highly correlated with cardiorespiratory function (Hills et al., 2024). Therefore, cardiorespiratory functions were emphasized in this study, and the differences in HRV and respiratory indices among those with different years of fitness Qigong training were examined.

HRV is increasingly used as an autonomic nervous modulation indicator (Agorastos et al., 2023; Lu and Kuo, 2014). Decreased HRV is an index of poor autonomic nervous system regulation, which is also associated with an increased risk of all-cause mortality (Agorastos et al., 2023). One recent Systematic Review and Meta-Analysis supports the role of structured exercise as an effective nonpharmacological strategy to enhance autonomic cardiovascular function, reflected by improvements in HRV, in older adults, with potential implications for reducing cardiovascular risk and promoting healthy aging (Etayo-Urtasun et al., 2025). The present study revealed index-specific group differences in HRV responses to a single session of fitness Qigong. Significant Group × Time interactions were found for SDNN, RMSSD, and LF, indicating that experience modulated dynamic HRV changes across exercise phases. The experienced group exhibited slightly higher SDNN at rest and markedly higher RMSSD during recovery compared with the inexperienced group, suggesting stronger parasympathetic reactivation and better autonomic adaptability following exercise. During recovery, significantly higher RMSSD in the experienced group, indicating stronger vagal reactivation following exercise. Consistent with previous findings, trained individuals typically show faster and/or greater early post-exercise vagal reactivation, reflected in elevated LnRMSSD during early recovery, compared with untrained adults (Dupuy et al., 2022). In the present study, SDNN decreased markedly during exercise and rebounded during recovery in both groups, consistent with the expected autonomic response to physical activity. Although the experienced group exhibited slightly lower SDNN values during exercise than the inexperienced group, this difference was not statistically significant. This finding aligns with the findings of Duan (Duan et al., 2025), who reported that competitive Tai Chi practitioners had higher resting SDNN but a larger reduction during Tai Chi compared to untrained students. Nevertheless, these findings in SDNN, RMSSD, and LF are consistent with previous research showing that fitness Qigong practice can enhance autonomic balance and promote physical fitness (Fong et al., 2015; Sun et al., 2024). According to HRV parameters, fitness Qigong exercise may play a role in promoting physical fitness. Even a 5-min fitness Qigong practice can trigger the elderly’s transient increase in vagal tone and concomitant decrease in sympathetic tone (Fong et al., 2015). The higher resting SDNN observed in the experienced group in our study supports the notion that long-term Qigong training enhances vagal modulation. Some of the possible reasons that account for this result are as follows: First, the movement of fitness Qigong–Yijinjing has obvious rhythm changes; that is, it first involves relaxation, tightening, and then loosenin (Larkey et al., 2024). Long-term fitness Qigong training with more rhythm changes in movement can improve the ability of conversion ability between sympathetic and vagal nerves, improve the function of the autonomic nervous system, make the excitability of the parasympathetic nerve dominant, and finally affect the change in HRV, reduce blood pressure, and slow down the heart rate (Larkey et al., 2024; Wang Z. et al., 2024; Zhu et al., 2020). Second, long-term fitness Qigong training can enhance the practice of breathing regulation with changes in breathing mode and depth, thereby increasing HRV (Feng et al., 2020). Finally, long-term fitness Qigong training may alleviate stress through mental training, which contributes to an increase in HRV (Fong et al., 2013; Kim et al., 2021).

No significant Group × Time interactions but significant time effects were found for HF and LF/HF, all showing expected decreases during exercise and partial recovery thereafter. These results suggest that both groups experienced the typical autonomic shift during exercise, characterized by sympathetic dominance, followed by vagal reactivation during recovery. Future studies should integrate measures such as respiratory rhythm, electromyographic activity, and long-term training adaptation to further clarify how expertise influences HRV regulation before, during, and after Fitness Qigong.

Abdominal respiration, characterized by deep diaphragmatic contractions that generate slow and voluminous breaths, is known to trigger the body’s relaxation response and is commonly recommended as a supportive therapy for cardiopulmonary and stress-related conditions (Chin and Kales, 2019; Gerritsen and Band, 2018; Shao et al., 2024). In Fitness Qigong, this breathing technique is intentionally practiced to promote mind–body harmony through controlled, deliberate respiration (Chen et al., 2019; Song et al., 2024). Overall, our study found that experienced Qigong practitioners breathed more deeply and exhibited lower breathing rates than inexperienced participants, as demonstrated by the main group effects. These findings align with the nature of the Qigong forms used (Yijinjing and Liuzijue) that emphasize slow-flowing movements synchronized with controlled breathing (Li et al., 2023). Existing studies have shown that Yijinjing or Liuzijue-based program can improve lung function and respiratory performance in clinical and healthy samples (Li et al., 2023; Zhang M. et al., 2016). For example, the randomized controlled trial by Zhang et al. in patients with chronic obstructive pulmonary disease demonstrated that 6 months of Yijinjing training significantly improved forced expiratory volume and forced expiratory volume in one second/forced vital capacity (Zhang M. et al., 2016). Our present findings also align with our earlier results, which showed that a 3-month Qigong intervention significantly increased abdominal breathing depth (*p* < 0.05), alongside improvements in HRV (SDNN) between groups (*p* = 0.039) (Sun et al., 2024). These findings support that long-term Qigong training could refine respiratory–autonomic coupling, enabling practitioners to maintain deeper, slower, and more stable respiration even under physical load. Moreover, we observed that the experienced group demonstrated greater abdominal respiration depth during exercise and slower thoracic respiration frequency during recovery. These results suggest that long-term Qigong training enhances respiratory efficiency by promoting coordinated diaphragmatic breathing during movement and facilitating slower, deeper breathing during recovery. Such adaptations have been associated with improved vagal tone and HRV (Lu and Kuo, 2014; Natarajan, 2023; Terathongkum and Pickler, 2004). Therefore, regular Fitness Qigong may serve as an effective, low-impact approach for enhancing respiratory function and autonomic regulation in middle-aged adults, both at rest and during moderate physical activity.

A previous study demonstrated that an effortless meditative state can lead to reduced sympathetic activity and increased vagal modulation (Park et al., 2022; Telles et al., 2013). Healthy individuals practicing meditation showed increased LF and HF components of HRV, and similar improvements were observed with Qigong and mindfulness in cancer survivors, indicating that meditative practices can enhance autonomic regulation and overall HRV (Lee Y.-H. et al., 2023; Nesvold et al., 2012). Therefore, meditative regulation that is produced during fitness Qigong exercise might also improve HRV indexes. The possible reasons that may account for this conjecture are as follows. First, meditation reduces habitual responses, thereby affecting motor processes and emotional control (Aftanas and Golosheykin, 2005; Tsang et al., 2025). Second, meditation evokes a relaxation response to improve mental health (Dal Lin et al., 2018; Tisdell et al., 2024). This response has been proven to be effective in improving depression and reducing stress (Goyal et al., 2014).

Although the preliminary results of the present study are promising, there are several limitations to this study. First, although the purpose of this study was to explore the effects of duration of fitness Qigong training on HRV, preliminary results were obtained from a cross-sectional study of only two groups of individuals who had practiced fitness Qigong for a long time and those who had just undergone fitness Qigong exercise, a longitudinal intervention study could be conducted in a follow-up study. Second, HRV can be influenced by multiple physiological and psychological states simultaneously, and a comprehensive assessment of participants’ psychological indicators and cognitive status can be conducted in future studies. Third, the participants selected for this study were overall healthy middle-aged adults. Therefore, our findings may not be generalizable to all people in this age group, and the effect of fitness Qigong on HRV in patients with certain medical conditions, such as frailty or metabolic syndrome, can be considered in future studies. Finally, HRV indices may be correlated with different durations and different types of fitness Qigong.

## 5 Conclusion

This study suggested that prolonged duration of Qigong exercise has a significant improvement in HRV, which is achieved by adjusting breathing in combination with mental concentration and relaxation, all of which ultimately contribute to health promotion.

## Data Availability

The raw data supporting the conclusions of this article will be made available by the authors, without undue reservation.

## Abbreviations

HRV, Heart rate variability; SDNN, Standard deviation of NN intervals; RMSSD, Root mean square of the successive differences; LF, Low frequency; HF, High frequency; LF/HF, Low frequency/High frequency ratio; SF-36, 36-Item Short Form Survey; BMI, Body mass index; BMR, Basal metabolic rate.
